# Cardiovascular Alterations and Management of Patients With White Coat Hypertension: A Meta-Analysis

**DOI:** 10.3389/fphar.2020.570101

**Published:** 2020-09-17

**Authors:** Huaqiang Xiang, Yangjing Xue, Jinsheng Wang, Yingbei Weng, Fangning Rong, Yangpei Peng, Kangting Ji

**Affiliations:** Department of Cardiology, The Second Affiliated Hospital and Yuying Children’s Hospital, Wenzhou Medical University, Wenzhou, China

**Keywords:** white coat hypertension, sustained hypertension, organ damage, cardiovascular risk, treatment

## Abstract

A large and growing body of literature has focused on the association between “white coat hypertension” (WCH) and the underlying target organ damage. The evidence suggests that WCH is may not an entirely benign phenomenon. However, whether patients with WCH should receive antihypertensive drugs is unresolved. Therefore, we performed a meta-analysis to fully determine the ability of WCH to alter cardiovascular structure and to determine whether patients with WCH could benefit from drug intervention. Medline, EMBASE, and the Cochrane Library were searched from inception through 21 Oct 2019. A total of 25 studies (8,100 individuals) were included. In participants with WCH, values of aortic pulse wave velocity, augmentation index, intima–media thickness, interventricular septum thickness, left ventricular posterior wall thickness, and left ventricular mass index were lower than those with sustained hypertension, but greater than those in the normotensive group. Of note, antihypertensive drug therapy did not reduce the risk of cardiovascular events in patients with WCH. WCH is accompanied by alterations of cardiovascular structure; however, the benefits from antihypertensive therapy are limited.

## Introduction

The term “white coat hypertension” (WCH) is used to describe untreated subjects whose blood pressure is elevated in the office, but is normal when measured by ambulatory blood pressure monitoring(ABPM) (Mancia and Zanchetti, 1996). Recent studies have shown that, compared with normotensive individuals, patients with WCH are more likely to suffer asymptomatic cardiac and vascular damage (Ihm et al., 2009; Fukuhara et al., 2013). A meta-analysis (Cuspidi et al., 2015) came out a similar result by comparing the value of intima–media thickness in subjects with or without WCH, regrettably, they did not involve other vascular or cardiac indexes. As we know, no study has comprehensively evaluated the association between WCH and the cardiac and vascular alterations. Furthermore, it remains unknown whether patients with WCH would benefit from antihypertensive treatment. Therefore, in the present study, we conducted a network meta-analysis to determine how WCH affects individuals by obtaining the value of ultrasonographic parameters in three groups (white coat hypertension, sustained hypertension, and normotension) who had not received treatment before and we compared the cardiovascular risk in subjects with or without medical treatment as well.

## Methods

### Search Strategy and Selection Criteria

We searched Medline, EMBASE, and the Cochrane Library from inception to 21 Oct 2019 using the terms: “White Coat Hypertension,” “Isolated Clinic Hypertension,” “White Coat Syndrome,” “isolated office hypertension,” and “White Coat effect.” We manually searched for additional eligible studies in reference lists of retrieved publications and relevant meta-analyses in the discipline. Studies were included if the patients they enrolled met the following criteria: 1) never received hypertension treatment before; 2) no clinical or laboratory evidence of coexisting cardiovascular disease; and 3) no clinical signs of secondary hypertension. Two reviewers (HX and YX) independently screened titles and abstracts based on inclusion criteria. After eliminating irrelevant studies, full text reports were reviewed. Case studies and review articles were excluded. Subsequently, we performed a manual search of all included cohort studies until no further relevant studies were identified. Disagreements between the two reviewers were resolved by a third reviewer (Ji Kangtin).

### Definition

We defined normotension (NT) as a consistently normal BP on both clinic blood pressure (CBP) and 24-h ambulatory blood pressure (ABP) measurements (CBP <140/90 mmHg and 24-h ABP <135/85 mmHg). WCH was defined as elevated CBP in the presence of normal 24-h ABP (CBP >140/90 mmHg and 24-h ABP <135/85 mmHg). Sustained hypertension (SH) was defined as both elevated CBP and 24-h ABP (CBP >140/90 mmHg and 24-h ABP >135/85 mmHg). Different cutoff points were also considered for eligibility. We selected three ultrasonographic parameters about angioarchitecture, which were aortic pulse wave velocity (PWV), augmentation index (AIX), and intima–media thickness (IMT). Meanwhile, interventricular septum thickness (IVST), left ventricular posterior wall thickness (PWT), left ventricular mass index (LVMI), left ventricular end-diastolic dimension (LVEDD), left ventricular end-systolic dimension (LVESD), early-to-late mitral flow velocity ratio (E/A), left ventricular fractional shortening (LVFS), and Ejection fraction (EF) were selected as parameters of cardiac structure. The cardiovascular events included sudden death, fatal myocardial infarction, heart failure, stroke, transient cerebral ischemic attack, angina pectoris, coronary revascularization, dissecting aortic aneurysm, limb claudication confirmed by angiogram, and carotid artery stenosis.

### Data Extraction

Two investigators (HX and YX) independently reviewed the reports and supplementary materials and extracted information into an electronic database: study and patient characteristics, thresholds for diagnosing WCH, study design, cardiac and vascular alterations, interventions, cardiovascular events, and duration of follow-up. Discrepancies regarding the extraction of data were resolved by a third investigator (KJ).

### Quality Assessment

The methodological quality of the cross-section studies included was assessed using an 11-item checklist which was recommended by Agency for Healthcare Research and Quality (AHRQ). An item would be scored “0” if it was answered “NO” or “UNCLEAR”; if it was answered “YES,” then the item scored “1.” Article quality was assessed as follows: low quality = 0–3; moderate quality = 4–7; high quality = 8–11.

The Newcastle-Ottawa Scale was used for the quality assessment of cohort studies. This scale appoints a maximum of nine stars to each study: four stars for the adequate selection of the two groups, two stars for comparability of groups on the basis of the design and analysis, and three stars for the adequate ascertainment of the exposure in both groups. High quality studies received nine stars and medium quality studies seven or eight stars. Detailed data are presented in **Tables 1A**, **B**.

**Table 1A:** Demographic characteristics and clinical parameters of the study population taking part in vascular alterations.

First Author, Year	Criteria of WCH	Groups	N	Ages (yrs)	Sex (M/F)	BMI	Smoke (%)	Glucose(mg/dl)	Cholesterol(mg/dl)	ABP (mmHg)	CBP (mmHg)
Zakopoulos et al., 1999	CBP >160/90 mmHg ASBP <130 mmHg	WCH	22	51.2± 13.3	10/12	–	50%	93.9 ± 1.8	222.3 ± 18.1	122.1 ± 5.7/75.5 ± 6.8	161.08 ± 1.35/96.6 ± 1.08
		NT	17	51.2± 12.8	11/6	–	41.20%	90.6 ± 0.4	212.18 ± 28.5	118.41 ± 6.09/72.29 ± 5.02	115.46 ± 0.38/75.02 ± 0.31
		SH	41	52.6 ± 10.8	21/20	–	46.30%	93.6 ± 2.0	215.95 ± 19.3	145.50 ± 9.9/89.04 ± 10	162.8 ± 0.38/103.0 ± 0.84
Nakashima et al., 2004	CB >140/90 mmHg ABP <135/85 mmHg	WCH	30	58 ± 9	10/20	23 ± 3	36.70%	98 ± 12	209 ± 37	129 ± 7/78 ± 9	157 ± 15/90 ± 10
		NT	30	58 ± 15	10/20	23 ± 3	33.30%	99 ± 23	214 ± 49/	126 ± 9/74 ± 8	130 ± 9/75 ± 9
		SH	30	54 ± 13	10/20	25 ± 4	36.70%	93 ± 12	224 ± 39	151 ± 12/85 ± 17	154 ± 16/90 ± 12
Manios et al., 2016	CSBP >140 mmHg CDBP >90 mmHg ABP <130/80 mmHg	WCH	228	55 ± 9	73/155	29 ± 5	31%	–	–	120 ± 7/73 ± 5	155 ± 13/97 ± 7
		NT	223	55 ± 11	100/123	26 ± 4	35%	–	–	113 ± 9/70 ± 6	121 ± 10/76 ± 7
		SH	666	55 ± 10	386/280	28 ± 4	39%	–	–	138 ± 11/84 ± 12	156 ± 18/97 ± 11
Kamel et al., 2006	CBP >140/90 mmHg ABP <135/85 mmHg	WCH	33	46 ± 7	18/15	29.6 ± 7.2	39%	96 ± 9.7	194 ± 34	117.7 ± 6.9/74.0 ± 6.4	141.3 ± 9.1/92.8 ± 7.2
		NT	17	45 ± 6	9/8	30.3 ± 4.4	41%	94 ± 6.8	188 ± 39	112.3 ± 7.6/71.4 ± 6.6	114.4 ± 10.9/72.3 ± 7.5
		SH	17	51 ± 10	8/9	30.2 ± 5.2	37%	95 ± 8.9	211 ± 26	136.8 ± 11.1/88.0 ± 5.3	156.4 ± 21.1/100.7 ± 7.6
Muldoon et al., 2000	CBP >140/90 mmHg ABP <140/90 mmHg	WCH	40	57 ± 10	40/0	28.5 ± 2.6	–	95.4 ± 18	210.1 ± 27.8	129 ± 7/80 ± 5	141 ± 10/90 ± 6
		NT	40	56 ± 9	40/0	26.8 ± 2.6	–	90 ± 9	199.2 ± 33.6	128 ± 7/80 ± 6	123 ± 9/80 ± 5
		SH	40	54 ± 10	40/0	28.5 ± 3.8	–	91.8 ± 7.2	206.9 ± 40.2	150 ± 9/89 ± 7	143+9/90 ± 6
Shih-Hsien et al., 2013	CBP >140/90 mmHg ABP <135/85 mmHg	WCH	153	58 ± 13	78/75	25 ± 4	–	104.4 ± 41.4	166.3 ± 42.5	122 ± 7/76 ± 5	145 ± 13/86 ± 9
		NT	250	48 ± 13	119/131	23 ± 3	–	95.4 ± 21.6	135.3 ± 34.8	110 ± 8/70 ± 6	107 ± 7/68 ± 6
		SH	536	55 ± 12	308/228	26 ± 4	–	104.4 ± 30.6	170.1 ± 50.2	143 ± 14/91 ± 9	155 ± 20/94 ± 12
Androulakis et al., 2016	CBP >140/90 mmHg ABP <135/85 mmHg	WCH	204	54.3 ± 0.9	95/109	28.5 ± 0.6	37.40%	99.4 ± 1.1	216. ± 3.1	120.1 ± 0.5/71.4 ± 0.5	152.9 ± 1.0/92.9 ± 0.9
		NT	183	52.4 ± 0.9	72/111	27.4 ± 0.6	42.90%	96.6 ± 0.9	212. ± 3.6	113.9 ± 0.5/67.8 ± 0.6	131.3 ± 1.0/83.1 ± 0.9
Neil et al., 2007	CBP >140/90 mmHg ABP <135/85 mmHg	WCH	18	51.4 ± 3.9	13/5	28.7 ± 2	–	–	–	–	–
		NT	18	41.9 ± 2.1	14/4	26.3 ± 1.3	–	–	–	–	–
Vyssoulis et al., 2010	CBP >140/90 mmHg ABP <135/85 mmHg	WCH	480	52.4 ± 0.6	196/284	27.2 ± 0.2	36.70%	–	211.1 ± 1.7	127.2 ± 0.7/83.6 ± 0.4	157.6 ± 0.4/97.8 ± 0.3
		NT	231	45.8 ± 1.0	119/112	26.2 ± 0.3	37.70%	–	199.5 ± 2.5	113.5 ± 0.8/76.5 ± 0.6	129.3 ± 0.5/82.0 ± 0.3
		SH	1188	52.4 ± 0.3	713/475	28.2 ± 0.1	41.20%	–	210.7 ± 1.1	138.4 ± 0.5/90.8 ± 0.3	164.7 ± 0.3/100.9 ± 0.2
Andrikou et al., 2011	CBP >140/90 mmHg ABP <135/85 mmHg	WCH	81	52 ± 8	51/30	28.9 ± 4	55%	95.9 ± 8.7	223 ± 37	119 ± 7/74 ± 11	146 ± 10/91 ± 8
		NT	44	52 ± 5	27/17	28.8 ± 5	49%	95.4 ± 7.3	220 ± 18	115 ± 8/74 ± 11	122 ± 8/87 ± 8
		SH	178	50 ± 8	119/59	29.3 ± 4	52%	97.6 ± 8	223 ± 36	148 ± 10/89 ± 7	152 ± 14/98 ± 8
Longo et al., 2006	CSBP: 140–159 mmHg CDBP: 90–99 mmHg ABP <135/85 mmHg	WCH	117	40.0 ± 0.8	76/41	24.1 ± 0.3	16.20%	91.8 ± 1.08	197.2 ± 4.25	124.7 ± 0.7/78.3 ± 0.5	143.1 ± 0.9/92.1 ± 0.5
		NT	51	36.8 ± 1.1	33/18	23.9 ± 0.4	37.30%	88.2 ± 0.9	189.5 ± 5.4	116.2 ± 1.3/71.3 ± 1.1	122.2 ± 1.5/71.9 ± 1.1
		SH	177	37.7 ± 0.7	128/49	25.0 ± 0.3	24.10%	93.6± 0.9	193.4 ± 3.09	138.9 ± 0.6/85.1 ± 0.6	146.6 ± 0.8/92.0 ± 0.5

BMI, body mass index; ABP, 24-h ambulatory blood pressure; CBP, clinical blood pressure; SABP, 24-h ambulatory systolic blood pressure WCH, white coat hypertension;

NT, normotension; SH, sustained hypertension; CSBP, clinical systolic blood pressure; CDBP, clinical diastolic blood pressure.

**Table 1B:** Demographic characteristics and clinical parameters of the study population taking part in cardiac alterations.

First Author, Year	Criteria of WCH	Groups	N	Ages (years)	Sex (M/F)	BMI	Smoke (%)	Glucose(mg/dl)	Cholesterol(mg/dl)	ABP (mmHg)	CBP (mmHg)
Grandi et al., 2001	CBP >140/90 mmHg ABP <130/80 mmHg	WCH	42	42 ± 7	18/24	–	–	–	–	120 ± 5/70 ± 5	154 ± 16/93 ± 14
		TN	42	42 ± 6	18/24	–	–	–	–	119 ± 6/71 ± 6	124 ± 5/73 ± 7
		SH	42	42 ± 7	18/24	–	–	–	–	144 ± 13/92 ± 8	153 ± 15/93 ± 12
Kuwajima et al., 1993	CBP >160/90 mmHg SABP <140 mmHg	WCH	17	74.1 ± 8.5	3/14	–	–	–	–	132.9 ± 6.3/74.5 ± 6.0	176.2 ± 11.7/91.1 ± 8.1
		TN	16	74.1 ± 6.1	5/11	–	–	–	–	124.8 ± 12.0/69.2 ± 5.6	133.8 ± 12.5/73.7 ± 11.2
		SH	34	73.6 ± 6.3	12/22	–	–	–	–	156.9 ± 10.6/84.3 ± 8.5	177.2 ± 14.9/89.6+11.1
Yildirim et al., 2012	CBP >140/90 mmHg ABP <135/85 mmHg	WCH	24	45.5 ± 13.2	7/17	29.2 ± 3.2	–	–	–	121.0 ± 8.16/74.5 ± 6.66	144.2 ± 4.58/90.8 ± 4.81
		TN	24	45.5 ± 9.6	7/17	29.9 ± 2.9	–	–	–	120.9 ± 7.10/76.3 ± 5.54	122.7 ± 8.21/76.5 ± 5.61
		SH	24	43.2 ± 9.8	8/16	30.0 ± 3.1	–	–	–	143.3 ± 9.17/90.2 ± 7.98	154.8 ± 12.29/96.5 ± 7.44
Pose-Reino et al., 1996	CBP >140/90 mmHg ABP <135/85 mmHg	WCH	27	46.3 ± 12	14/13	27.5 ± 3.2	–	–	–	120 ± 8/71 ± 6	148 ± l1/96 ± 5
		TN	51	42.7 ± 11.3	27/24	26.6 ± 3	–	–	–	110 ± 5/65.7 ± 5	125 ± 9/79 ± 6
		SH	24	46.7 ± l5	11/13	27.5 ± 3.1	–	–	–	135 ± 10/79 ± 7	162 ± 15/97 ± 6
Torris et al., 1999	CBP >160/95 mmHg ABP <135/85 mmHg	WCH	29	72.4 ± 4.9	14/15	25.8 ± 2.9	–	–	–	133.3 ± 7.2/77.9 ± 7.1	164.4 ± 9.99/83.6 ± 6.8
		TN	33	69.9 ± 4.5	16/17	25.1 ± 2.6	–	–	–	118 ± 3.2/73.4 ± 7.8	124.3 ± 17.8/79 ± 7.5
		SH	87	71.4 ± 4.9	46/41	26.5 ± 2.8	–	–	–	153 ± 17.4/85.6 ± 12	174.2 ± 45.5/94.4 ± 12.7
Andrikou et al., 2011	CBP >140/90 mmHg ABP <135/85 mmHg	WCH	81	52 ± 8	51/30	28.9 ± 4	55%	95.9 ± 8.7	223 ± 37	119 ± 7/74 ± 11	146 ± 10/91 ± 8
		TN	44	52 ± 5	27/17	28.8 ± 5	49%	95.4 ± 7.3	220 ± 18	115 ± 8/74 ± 11	122 ± 8/87 ± 8
		SH	178	50 ± 8	119/59	29.3 ± 4	52%	97.6 ± 8	223 ± 36	148 ± 10/89 ± 7	152 ± 14/98 ± 8
Androulakis et al., 2016	CBP >140/90 mmHg ABP <135/85 mmHg	WCH	204	54.3 ± 0.9	95/109	28.5 ± 0.6	37.40%	99.4 ± 1.1	216 ± 3.1	120.1 ± 0.5/71.4 ± 0.5	152.9 ± 1.0/92.9 ± 0.9
		TN	183	52.4 ± 0.9	72/111	27.4 ± 0.6	42.90%	96.6 ± 0.9	212 ± 3.6	113.9 ± 0.5/67.8 ± 0.6	131.3 ± 1.0/83.1 ± 0.9
Ermis et al., 2016	CBP >140/90 mmHg ABP <130/80 mmHg	WCH	37	48.4 ± 5.7	17/20	25.3 ± 3.0	–	90.1 ± 12.4	203.1 ± 37.4	–	147.1 ± 7.2/93.2 ± 4.4
		TN	38	47.9 ± 7.5	18/20	26.5 ± 3.7	–	88.4 ± 11.7	203.2 ± 36.9	–	112.2 ± 9.3/72.8 ± 5.9
Sega et al., 2001	CABP >140 mmHg ABP <125 mmHg	WCH	178	58.5 ± 10.8	89/89	–	–	–	–	118.7 ± 5.6	149.0 ± 9.4
		TN	1090	44.3 ± 11.8	501/589	–	–	–	–	113.4 ± 6.7	111.9 ± 10.4
		SH	220	59.5 ± 9.7	130/90	–	–	–	–	137.4 ± 9.4	159.0 ± 15.2
Soma et al., 1996	CABP >140 mmHg ABP <140/90 mmHg	WCH	26	46 ± 13	12/14	–	–	–	–	122 ± 7/79 ± 6	159 ± 18/98 ± 5
		TN	32	48 ± 7	19/13	–	–	–	–	118 ± 13/77 ± 6	118 ± 13/77 ± 6
		SH	22	48 ± 7	9/13	–	–	–	–	141 ± 12/96 ± 6	159 ± 17/105 ± 5
Shih-Hsien et al., 2013	CBP >140/90 mmHg ABP <135/85 mmHg	WCH	153	58 ± 13	78/75	25 ± 4	–	104.4 ± 41.4	166.3 ± 42.5	122 ± 7/76 ± 5	145 ± 13/86 ± 9
		TN	250	48 ± 13	119/131	23 ± 3	–	95.4 ± 21.6	135.3 ± 34.8	110 ± 8/70 ± 6	107 ± 7/68 ± 6
		SH	536	55 ± 12	308/228	26 ± 4	–	104.4 ± 30.6	170.1 ± 50.2	143 ± 14/91 ± 9	155 ± 20/94 ± 12
Kamel et al., 2006	CBP >140/90 mmHg ABP <135/85 mmHg	WCH	33	46 ± 7	18/15	29.6 ± 7.2	39%	96 ± 9.7	194 ± 34	117.7 ± 6.9/74.0 ± 6.4	141.3 ± 9.1/92.8 ± 7.2
		TN	17	45 ± 6	9/8	30.3 ± 4.4	41%	94 ± 6.8	188 ± 39	112.3 ± 7.6/71.4 ± 6.6	114.4 ± 10.9/72.3 ± 7.5
		SH	17	51 ± 10	8/9	30.2 ± 5.2	37%	95 ± 8.9	211 ± 26	136.8 ± 11.1/88.0 ± 5.3	156.4 ± 21.1/100.7 ± 7.6
Sang-Hyun 2009	CSBP: 140–159 mmHg, CDBP: 90–99 mmHg, ABP <135/85 mmHg	WCH	30	48 ± 9	10/20	24 ± 3	–	–	–	–	–
		TN	30	47 ± 9	12/18	23 ± 3	–	–	–	–	–
		SH	30	51 ± 7	13/17	25 ± 3	–	–	–	–	–
Mustafa 2013	CBP >140/90 mmHg ABP <135/85 mmHg	WCH	40	45.1 ± 71	18/22	28.1 ± 2.2	–	94.2 ± 7.9	189.3 ± 30	115.9 ± 10.6/74.3 ± 10.3	146.2 ± 7.3/92.7 ± 4.3
		TN	39	44.8 ± 7.0	18/21	27.9 ± 2.2	–	92.7 ± 6.1	190.1 ± 27.3	116.3 ± 10.5/74.1 ± 10.4	123.8 ± 7.4/76.9 ± 5.8
		SH	62	45.1 ± 7.4	32/30	27.2 ± 2.9		95.5 ± 6.6	197.8 ± 27.2	136.7 ± 9.5/88.1 ± 7.4	146.6 ± 6.9/93.5 ± 4.6
Verdecchia 1992	CBP >90 mmHg ABP <134/90 mmHg	WCH	57	50 ± 10	24/33	–	–	–	–	125 ± 5/82+5	144 ± 12/93 ± 4
		TN	47	49 ± 9	27/20	–	–	–	–	127 ± 8/79 ± 7	127 ± 8/79 ± 7
		SH	289	52 ± 11	153/136	–	–	–	–	149 ± 13/97 ± 8	160 ± 18/98 ± 7

CBP, clinical blood pressure; CSBP, clinical systolic blood pressure; CDBP, clinical systolic blood pressure; ABP, 24-h ambulatory blood pressure; ASBP, 24-h ambulatory systolic blood pressure; WCH, white coat hypertension; NT, normotension; SH, sustained hypertension; BMI, body mass index.

### Data Analysis

We initially performed pairwise meta-analysis to calculate the relative risk (RR) or mean difference (MD) and their appropriate 95% confidence intervals (CIs) from original studies with methods described by Tierney and colleagues (Tierney et al., 2007). We pooled summary estimate using the Daimonian–Laird random-effects method (Dersimonian and Nan, 1986), which recognizes and anchors studies as a sample of all potential studies. The I^2^ statistic was calculated as a measure of the proportion of the overall variation that is attributable to between-study heterogeneity.

For indirect and mixed comparisons, we used network meta-analysis to compare the differences of cardiac and vascular structure in patients with WCH, NT, and SH. The results were expressed as mean difference (MD). Ranking probabilities for all BP phenotypes were estimated to obtain a hierarchy using the mean ranks. A loop-specific approach was used to evaluate the presence of inconsistency locally in network meta-analysis models, i.e., if the information from both sources of evidence (direct and indirect estimations) were similar enough to be combined. Inconsistency was defined as disagreement between direct and indirect evidence with a 95% CI. Analyses were conducted using Review Manger (version 5.1), STATA (version 13), and R software (version 3.6).

## Results

### Study Characteristics

**Figure 1:**
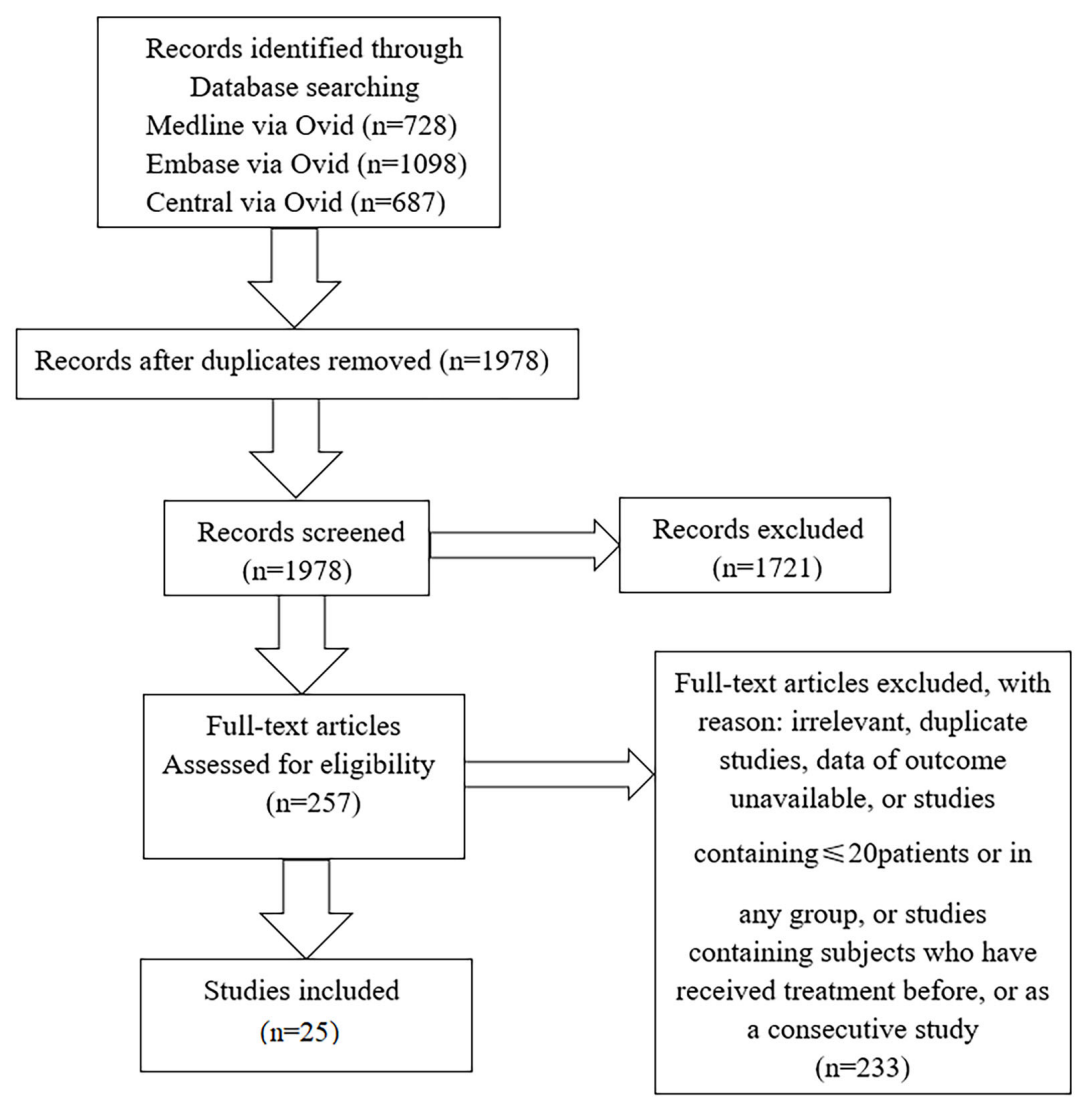
Flowchart of studies considered for inclusion.

We collected 25 studies, including 22 cross-sectional studies, two observational studies, and one randomized controlled trial. We classified articles into three categories based on research purpose. Eleven studies were concerned with changes of angioarchitecture and 15 studies were associated with carotid structural changes (four articles mentioned both alterations), and we collected three studies about treatment. The flowchart of the literature search was shown in **Figure 1**.

In studies of angioarchitecture, there were 1,406 participants with WCH, 1,104 participants with NT, and 2,873 participants with SH. There were no differences among groups with respect to age, sex, BMI, smoking status, or serum glucose levels; however, patients in the WCH and SH groups had higher levels of serum total cholesterol than those in the NT group (**Table 1A**).

In studies of cardiac structure, there were 978 participants with WCH, 1,936 participants with NT, and 1,565 participants with SH. Detailed data are presented in **Table 1B**. SH group had a higher proportion of male than the other two groups. And patients in the WCH and SH groups had higher levels of serum total cholesterol than those in the NT group.

In studies regarding treatment, there were 414 patients with WCH, 169 of whom received oral drug therapy, while the remaining 245 participants did not (**Table 1C**). The mean follow-up was 48 months. The two groups did not differ with respect to age, BMI, proportion of males, or 24-h systolic and diastolic BP.

**Table 1C:** Demographic characteristics and clinical parameters of the study population who received treatment or not.

First Author, Year	Criteria of WCH	Intervention Groups	N	Ages (yrs)	Sex (M/F)	BMI (Kg/m^2^)	Smokers(n)	Follow up(months)	ABP (mmHg)	CBP (mmHg)
Polonia et al., 2005	140/90 mmHg < CBP <175/105 mmHg ABP <130/85 mmHg	Treated	45	49 ± 2.1	17/28	24.8 ± 0.5	3	88	119.5 ± 1.6/73.6 ± 1.1	150.1 ± 0.9/93.1 ± 1.3
		Untreated	34	45.9 ± 2.5	14/20	24.5 ± 0.4	2		117.9 ± 1.6/73.8 ± 1.1	151.3 ± 1.5/92.2 ± 1.4
Hoshide et al., 2002	CBP >140/90 mmHg, ABP <130/80 mmHg	Treated	76	70 ± 9.6	30/46	24 ± 3.9	18	41	121 ± 6.5/69 ± 5.0	156 ± 12/83 ± 12
		Untreated	160	72 ± 9.9	50/110	24 ± 3.3	16		119 ± 8.1/69 ± 5.9	156 ± 10/83 ± 12
Fagard et al., 2000	160 mmHg < CSBP <219, CDBP <95 mmHg, ASBP <140 mmHg	Treated	48	69.3 ± 5.6	37/62	26.9 ± 4.3	–	11.7 ± 3.4	132.3 ± 6.0/78.5 ± 7.8	168.0 ± 6.5/86.0 ± 5.3
		Untreated	51							

CBP, clinical blood pressure; ABP, 24-h ambulatory blood pressure; BMI, body mass index; CSBP, clinical systolic blood pressure; CDBP, clinical systolic blood pressure; ASBP, 24-h ambulatory systolic blood pressure.

### Vascular Changes in Subjects With WCH, NT, and SH

**Figure 2:**
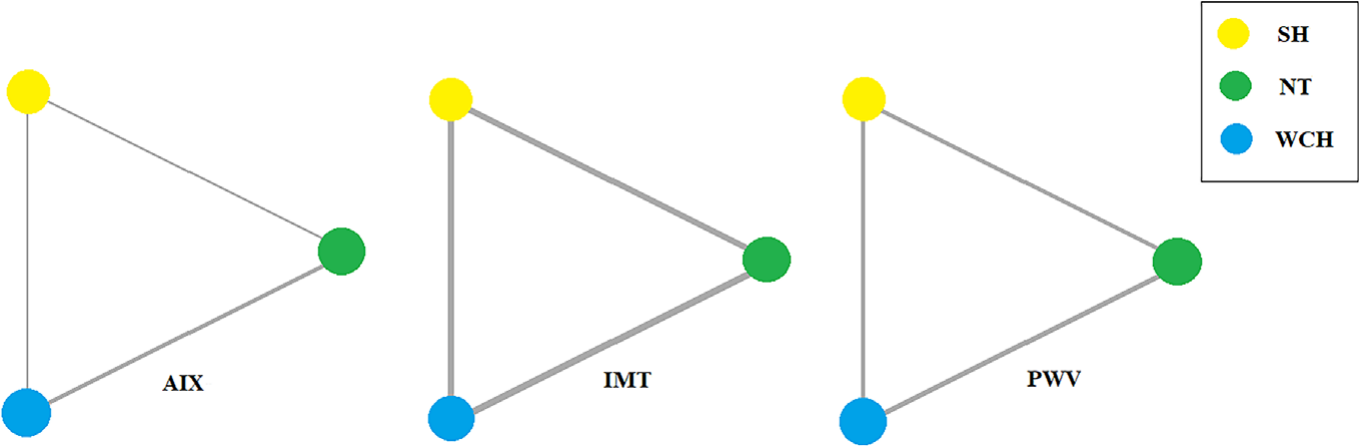
Evidence structure of eligible comparisons on alterations of angioarchitecture. SH, sustained hypertension; NT, normotension; WCH, white coat hypertension; AIX, augmentation index; IMT, intima–media thickness; PWV, aortic pulse wave velocity.

Network evidence on alterations of angioarchitecture is shown in **Figure 2**. The numbers along the link lines indicate the number of studies or pairs of study arms. The width of the lines represents the cumulative number of studies for each pairwise comparison. The size of every node is proportional to the number of participants.

**Figure 3:**
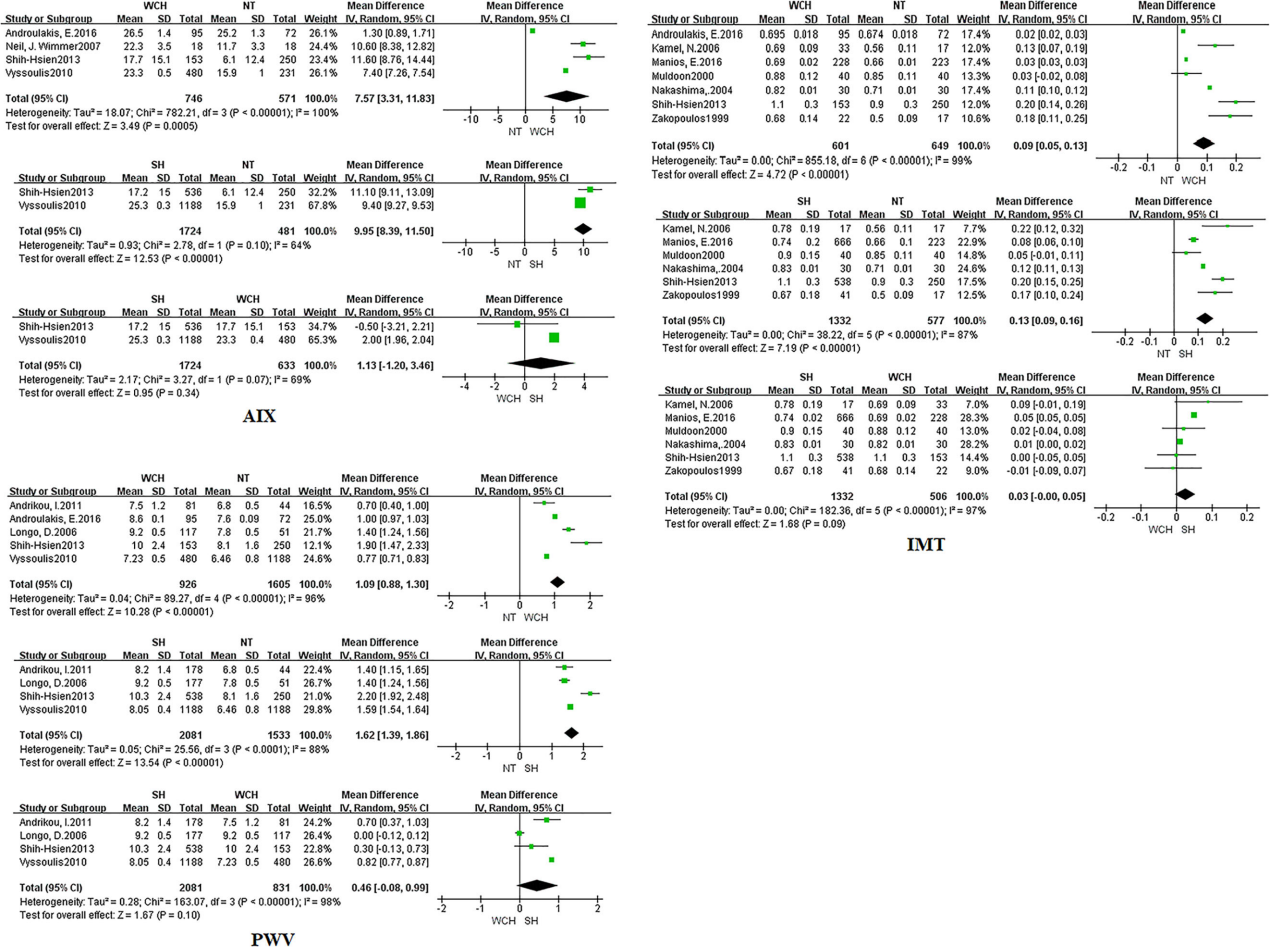
Different value of AIX, IMT, and PWV from pairwise meta-analyses. SH, sustained hypertension; NT, normotension; WCH, white coat hypertension; AIX, augmentation index; IMT, intima–media thickness; PWV, aortic pulse wave velocity.

In studies of angioarchitecture, nine trials were three-arm studies, and two were two-arm studies reporting data for NT and WCH. **Figure 3** shows the effect of WCH, NT, SH on vascular alterations from pairwise meta-analyses (direct meta-analysis). Compared with normal subjects, patients with WCH and SH had signiﬁcantly higher value of PWV, AIX, and IMT. In addition, patients with SH had higher values of IMT than patients with WCH.

**Figure 4:**
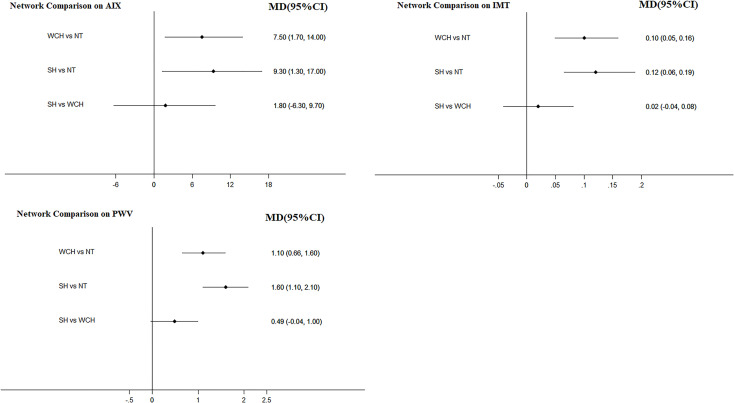
Effect of WCH, NT, and SH on vascular alterations from network meta-analysis. SH, sustained hypertension; NT, normotension; WCH, white coat hypertension; AIX, augmentation index; IMT, intima–media thickness; PWV, aortic pulse wave velocity.

The network meta-analysis is shown in **Figure 4**. Compared with normal subjects, patients with WCH and SH had significantly higher value of IMT, PWV, and AIX. Furthermore, there was no significantly difference between subjects with WCH and SH.

**Figure 5:**
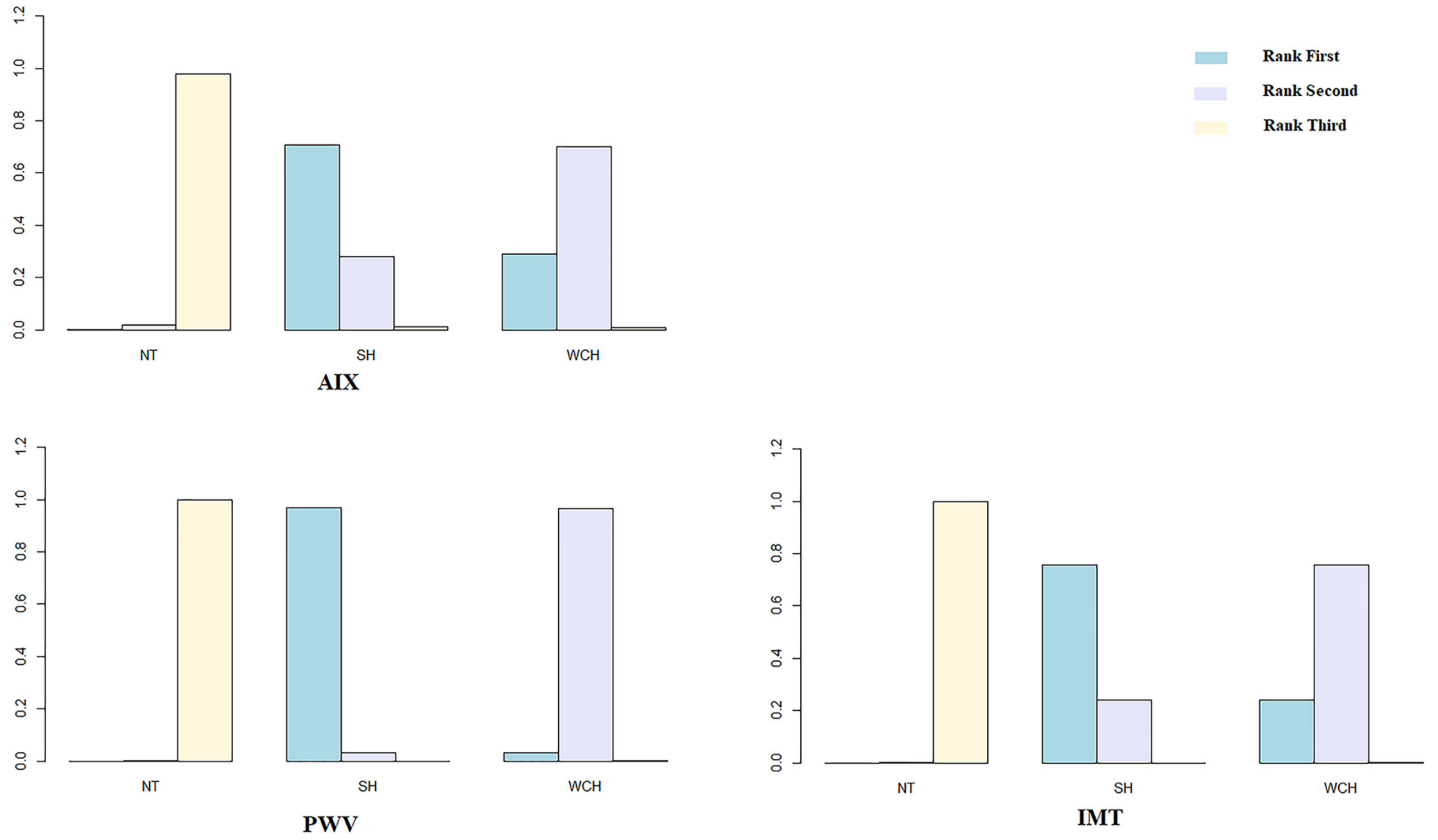
Ranking probability of each groups on vascular alterations. SH, sustained hypertension; NT, normotension; WCH, white coat hypertension; AIX, augmentation index; IMT, intima–media thickness; PWV, aortic pulse wave velocity.

**Figure 5** illustrates the ranking probability of each group in terms of angioarchitectural alterations. SH was most likely to rank first in AIX, PWV, IMT, which means that patients with SH have the highest values of those indexes. WCH ranked second, and NT ranked third. These findings suggest that alterations in angioarchitecture in WCH patients were intermediate between patients with SH and NT.

**Figure 6:**
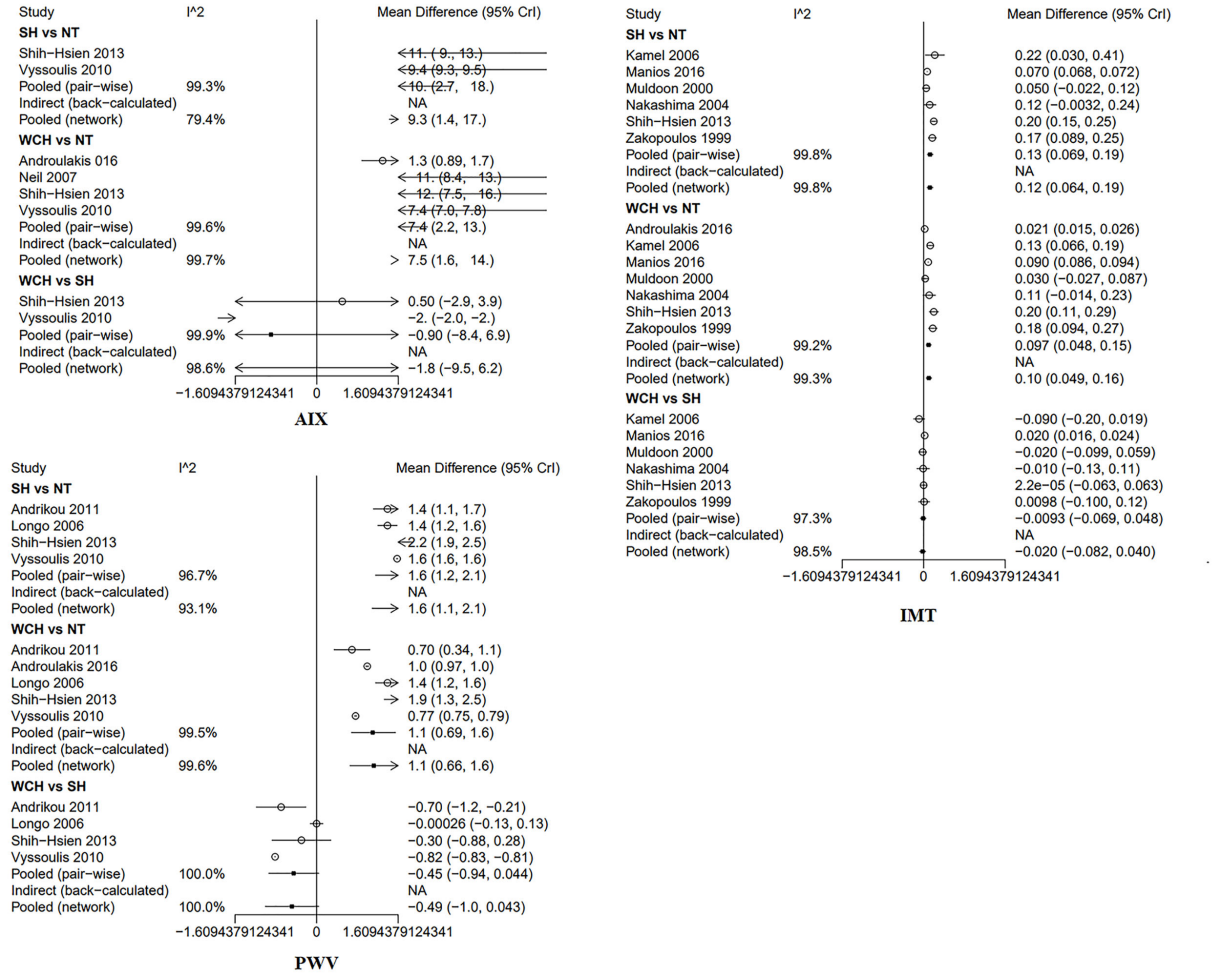
Inconsistence check in network. SH, sustained hypertension; NT, normotension; WCH, white coat hypertension; AIX, augmentation index; IMT, intima–media thickness; PWV, aortic pulse wave velocity.

As illustrated in **Figure 6**, when we directly compared these differences among the three BP phenotypes, the results obtained from pairwise meta-analyses and network meta-analyses were inconsistent.

### Alterations of Cardiac Structure Subjects With WCH, NT, and SH

**Figure 7:**
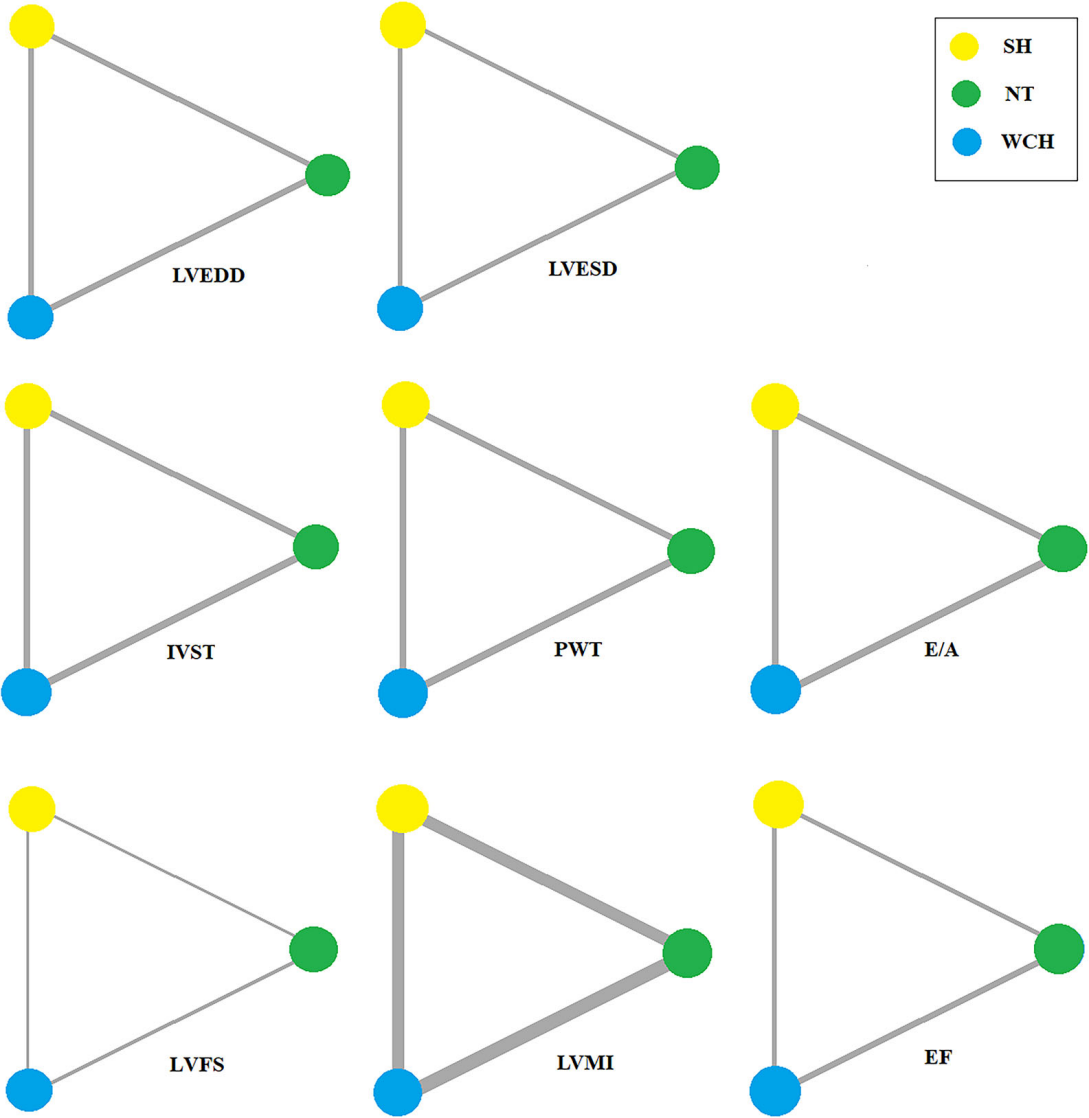
Evidence structure of eligible comparisons on alterations of cardiac structure. SH, sustained hypertension; NT, normotension; WCH, white coat hypertension; LVEDD, left ventricular end-diastolic dimension; LVESD, left ventricular end-systolic dimension; IVST, interventricular septum thickness; PWT, left ventricular posterior wall thickness; E/A, early-to-late mitral flow velocity ratio; LVFS, left ventricular fractional shortening; LVMI, left ventricular mass index; EF, ejection fraction.

In studies of cardiac structure, thirteen trials were three-arm studies and two were two-arm studies comparing data for NT and WCH groups. Network evidence on alterations of angioarchitecture are shown in **Figure 7**.

**Figure 8:**
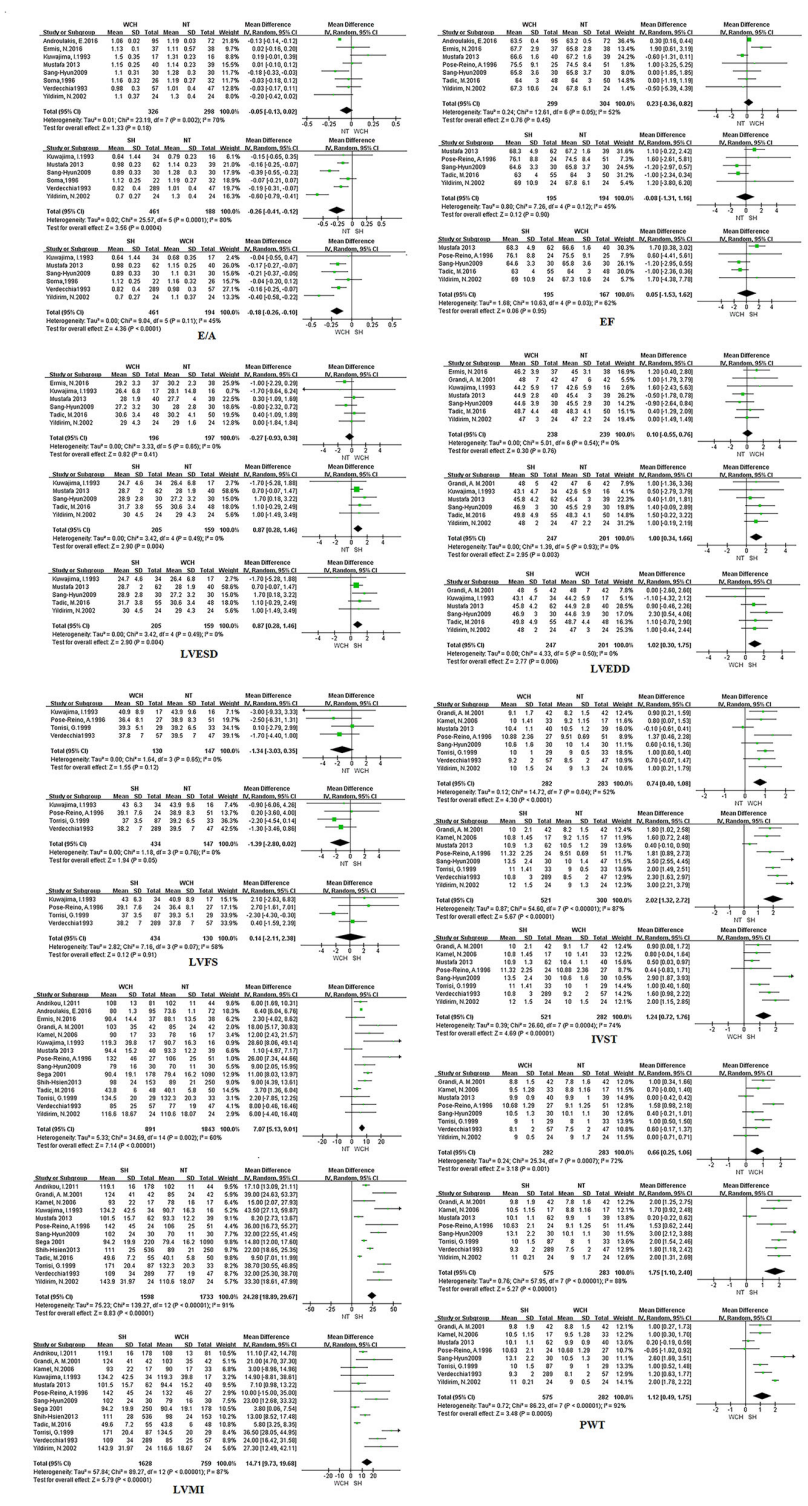
Different value of cardiac structure index from pairwise meta-analyses. SH, sustained hypertension; NT, normotension; WCH, white coat hypertension; LVEDD, left ventricular end-diastolic dimension; LVESD, left ventricular end-systolic dimension; IVST, interventricular septum thickness; PWT, left ventricular posterior wall thickness; E/A, early-to-late mitral flow velocity ratio; LVFS, left ventricular fractional shortening; LVMI, left ventricular mass index; EF, ejection fraction.

**Figure 8** shows the effect of WCH, NT, and SH on carotid structural changes using pairwise meta-analyses (direct meta-analysis). Patients with WCH and SH had signiﬁcantly higher value of IVST, PWT, and LVMI than normal subjects, meanwhile, patients with SH had greater value of IVST, PWT, and LVMI than patients with WCH. By contrast, there was no significantly different value of LVEDD, LVESD, E/A, LVFS, and EF between WCH and NT groups.

**Figure 9:**
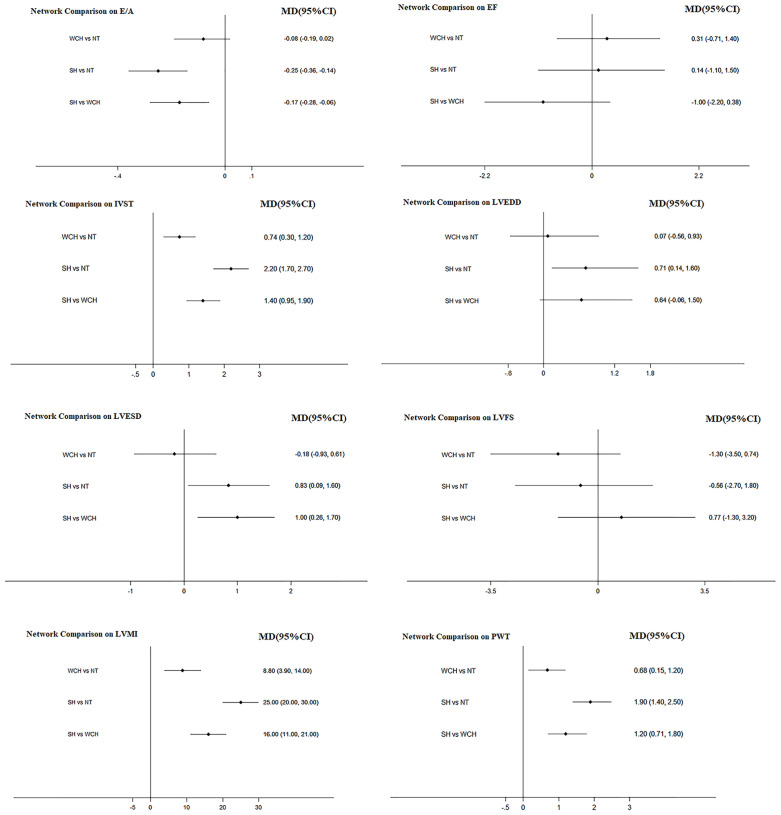
Effect of WCH, NT, and SH on cardiac alterations from network meta-analysis. SH, sustained hypertension; NT, normotension; WCH, white coat hypertension; LVEDD, left ventricular end-diastolic dimension; LVESD, left ventricular end-systolic dimension; IVST, interventricular septum thickness; PWT, left ventricular posterior wall thickness; E/A, early-to-late mitral flow velocity ratio; LVFS, left ventricular fractional shortening; LVMI, left ventricular mass index; EF, ejection fraction.

The result of network meta-analysis on cardiac structure alterations are shown in **Figure 9**. Patients with WCH and SH had significantly higher value of IVST, LVMI, and PWT than patients with NT. In addition, patients in the SH group had significantly higher value of IVST and PWT than patients in the WCH group, and there was no significantly different value of EF, E/A, LVEDD, LVESD, and LVFS between groups of WCH and NT.

**Figure 10:**
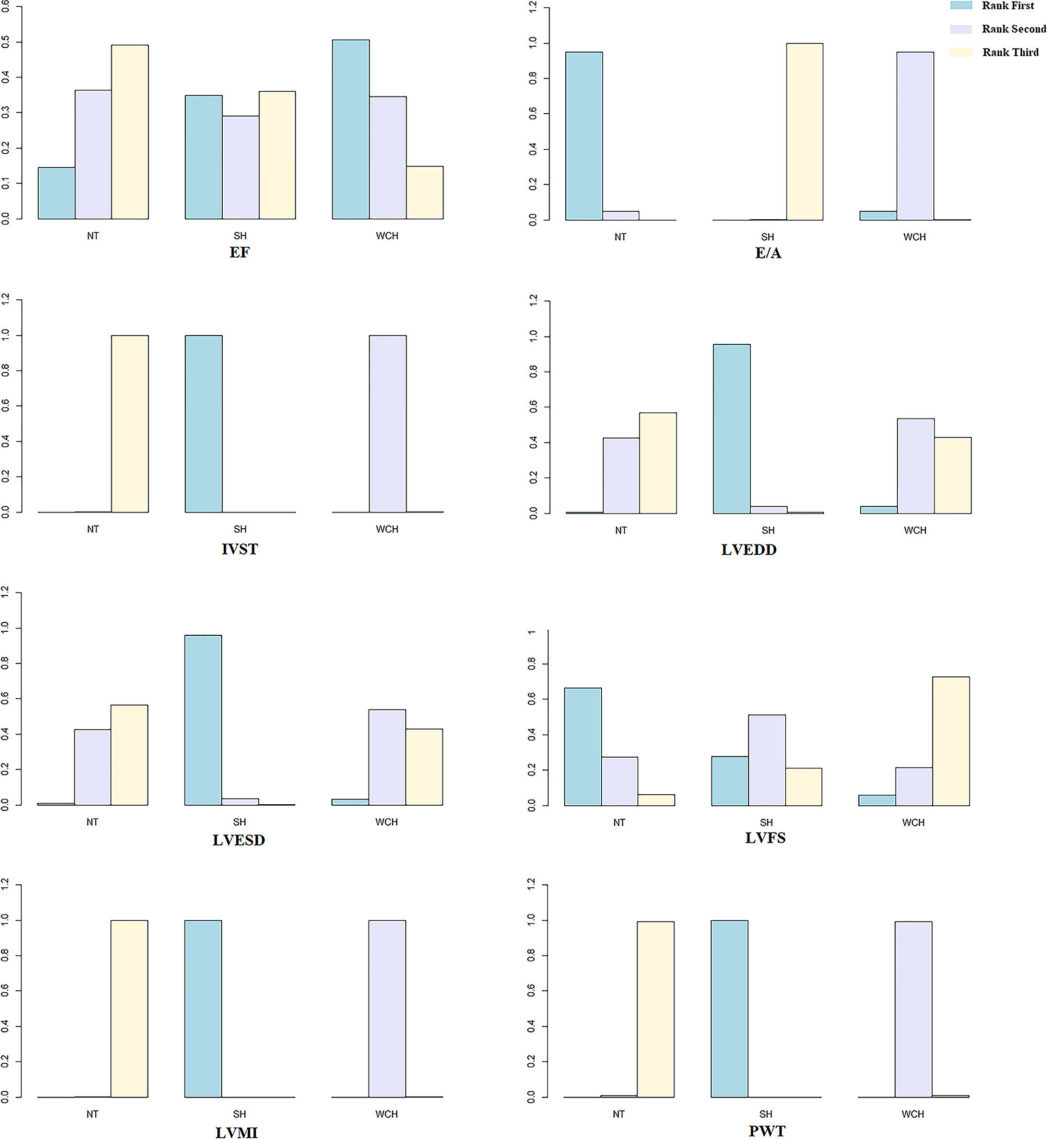
Ranking probability of each groups on cardiac alterations. SH, sustained hypertension; NT, normotension; WCH, white coat hypertension; LVEDD, left ventricular end-diastolic dimension; LVESD, left ventricular end-systolic dimension; IVST, interventricular septum thickness; PWT, left ventricular posterior wall thickness; E/A, early-to-late mitral flow velocity ratio; LVFS, left ventricular fractional shortening; LVMI, left ventricular mass index; EF, ejection fraction.

**Figure 10** shows the ranking probability of each groups with respect to carotid structural changes. SH ranked first in terms of LVEDD, LVESD, PWT, IVST, and LVMI; WCH ranked first in EF; NT ranked first in E/A, LVFS. The group ranking first had the highest value of those index.

**Figure 11:**
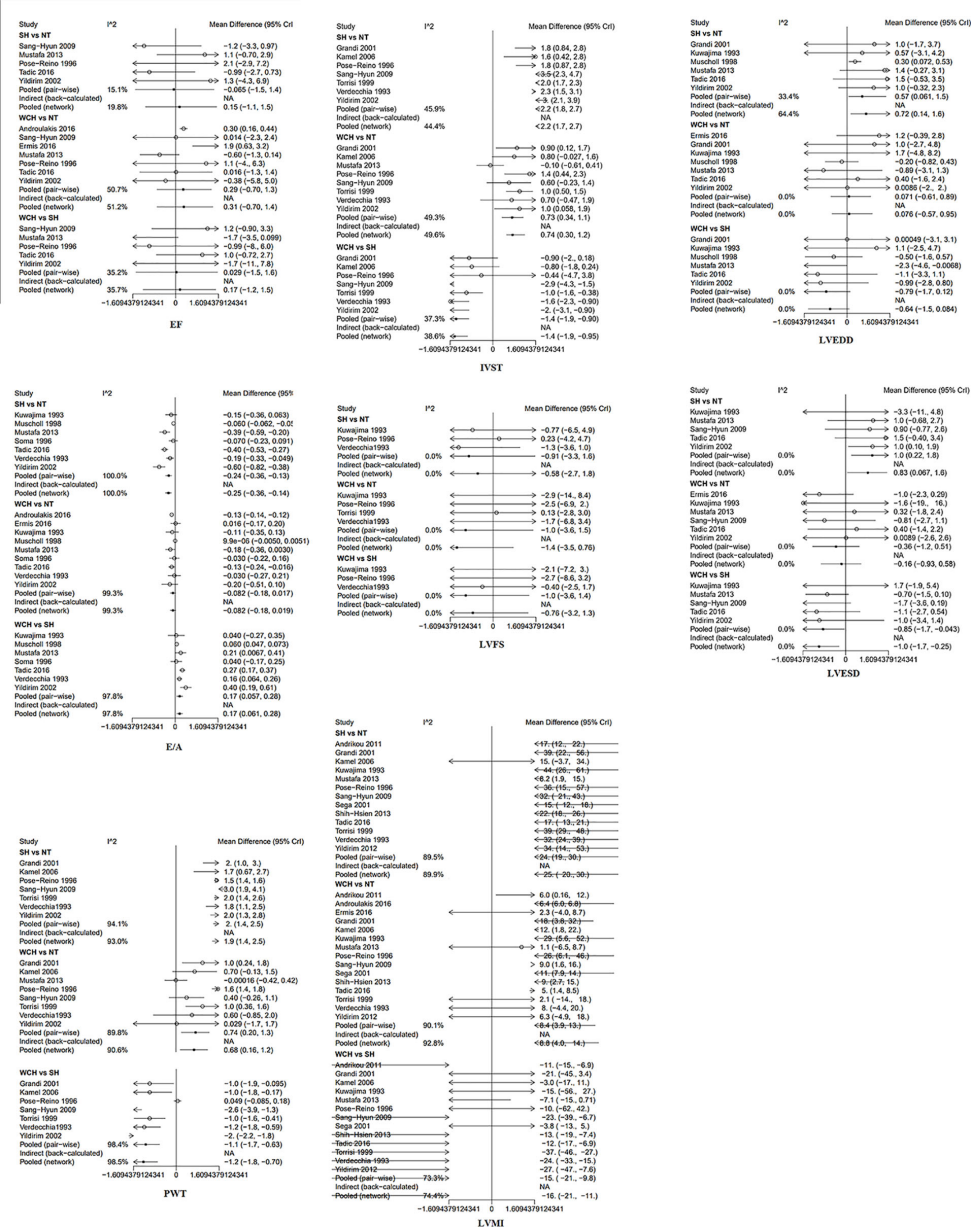
Inconsistence check in network. SH, sustained hypertension; NT, normotension; WCH, white coat hypertension; LVEDD, left ventricular end-diastolic dimension; LVESD, left ventricular end-systolic dimension; IVST, interventricular septum thickness; PWT, left ventricular posterior wall thickness; E/A, early-to-late mitral flow velocity ratio; LVFS, left ventricular fractional shortening; LVMI, left ventricular mass index; EF, ejection fraction.

As illustrated in **Figure 11**, when we directly compared parameters among the three BP phenotypes, the results obtained from pairwise meta-analyses were inconsistent with those of the network meta-analyses.

### Effect of Drug Therapy

**Figure 12:**
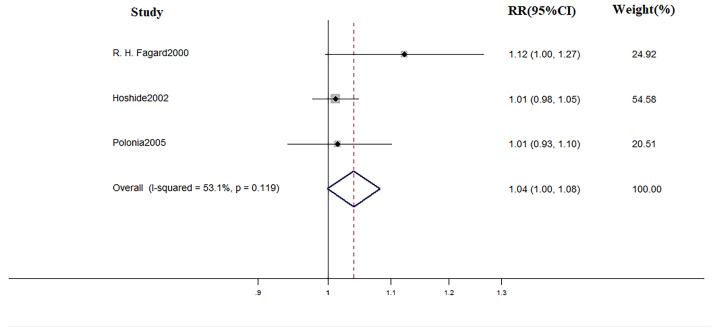
Different risk of cardiovascular events in WCH subjects with or with treatment.

In terms of treatment, during the mean follow-up of 48 months, there were 18 cardiovascular events, 14 of which occurred in WCH patients. The incidence of cardiovascular events did not differ in WCH subjects broken down by treatment status (**Figure 12**).

## Discussion

### Cardiac and Vascular Alterations in WCH Subjects

One of our main findings was that patients with WCH had greater values of the PWV, AIX, IMT, LVMI, PWT, IVST than normal subjects. O’Leary et al. (1999) reported that the increase in IMT was accompanied by higher risk of myocardial infarction and stroke in older adults. A meta-analysis reported that PWV was a strong predictor of future CV events and all-cause mortality (Vlachopoulos et al., 2010). Furthermore, Nürnberger found that the increase of AIX was associated with higher cardiovascular risk (Nürnberger et al., 2002). Taken together, the evidence suggests that elevation of these three indexes reflects the fact that patients with WCH may be at higher risk of cardiovascular events. Values of IVST, PWT, and LVMI, which are all criteria for left ventricular hypertrophy, were also significantly higher in patients with WCH. It has been demonstrated that left ventricular hypertrophy strongly predicts cardiovascular disease and its prognosis (Manyari, 1990).

The findings of the present study support the view that WCH does have a negative physiological effect on patients. Furthermore, the cardiovascular risk tends to be higher in these patients. Specifically, a recent meta-analysis (Jordana et al., 2019) comprising 25,786 individuals with untreated WCH indicated that compared to normotension, WCH was associated with a 1.36-fold risk of cardiovascular events with a mean duration of 3 to 19 years.

However, there was apparent heterogeneity in our result. The heterogeneity remained when we pooled results using random-effects models or excluded any of the studies. The following reasons may explain this discrepancy. First, differing demographic characteristics and clinical parameters in the study populations may result in inconsistent values. Second, case histories of the participants with abnormal blood pressure, which would have a substantial impact on the results, were not recorded in the original articles. Finally, the incorporation of studies using different criteria of WCH may account for the discrepancy. Because of the apparent heterogeneity across studies, the findings from our study should be interpreted with some caution.

### Treatment in WCH

Another main finding was that the effect of medical intervention in patients with WCH is probably limited. Some studies have drawn some conclusion. Bulpitt found that, among WCH patients older than 80 years, antihypertension drugs reduced the office BP with no concomitant reduction of ambulatory BP values (Bulpitt et al., 2013). Mania observed the same phenomenon in younger patients (Mancia et al., 2014). It is worth noting that ambulatory blood pressure is considered to be a better predictor than office BP of CV outcomes (Fagard et al., 2008; Roush et al., 2014; Banegas et al., 2018). In the present study, we took cardiovascular events as the end-point and medical intervention did not significantly decrease the CV risk.

There are several explanations for why treatment is not effective. Temporary elevated blood pressure may not be the main reason for the increased cardiovascular risk in patients with WCH. Because oral antihypertension medications lower office blood pressure, blood pressure in these patients should be normal all through the day (their out-of-office blood pressure was already normal).

We believe that sympathetic hyperactivity and metabolic disorders may contribute to the development of organ damage and cardiovascular events in patients with WCH. First, previous studies reported that WCH was associated with increased adrenergic activity that is not marginal but similar to that seen in SH (Smith et al., 2002; Grassi et al., 2007). Santulli explained the role of the pathophysiological and the sympathetic nervous systems in heart failure and cardiovascular aging (Santulli and Iaccarino, 2016). A review stated that sympathetic hyperactivity can exacerbate the harmful effects of preexisting cardiac ischemia (Shanks and Herring, 2013). These findings suggest that excessive activation of the nervous system may account for the rising CV risk. Second, subjects with WCH may have unfavorable metabolic profiles (Mancia et al., 2006).

Subjects with WCH tend to had higher levels of serum total cholesterol, and blood glucose and had higher BMI, all of which are risk factors for cardiovascular disease. Some studies found that the risk for coronary heart disease and stroke was increased twofold or threefold in subjects with metabolic syndrome (Isomaa et al., 2001; Mottillo et al., 2010). The increased cardiovascular risk in patients with WCH probably results from alterations of the endocrine and nervous systems that would likely decrease the effect of oral drugs. Overall, anti-hypertension medications do not benefit patients with WCH. In the outpatient clinic, we usually define hypertension by elevated office BP; however, in patients with an elevated office BP, WCH can account for 30–40% (and >50% in the very old) (Bryan et al., 2018). Therefore, it is important to make the correct diagnosis to avoid excessive medical treatment.

### Limitation

In addition to the heterogeneity among studies, several limitations were worthy of mention. First, the number of the studies about treatment was relatively small. Second, the criterion of WCH was relatively broad. Overall, more large randomized controlled studies are highly needed.

## Author Contributions

The role of each author: KJ made the conception and design of the review. HX and YX made the analysis and interpretation of the data as well as drafting the manuscript. JW, YP, FR, and YW made the critical revision of the manuscript for important intellectual content.

## Conflict of Interest

The authors declare that the research was conducted in the absence of any commercial or financial relationships that could be construed as a potential conflict of interest.
