# Supplementary material for: Cardiovascular Alterations and Management of Patients With White Coat Hypertension: A Meta-Analysis
Source: Front Pharmacol. 2020 Sep 17;11:570101. doi: 10.3389/fphar.2020.570101 (PMC7527598; doi:10.3389/fphar.2020.570101)
Supplement: Supplementary file 1 [file Table_1.docx]

| Supplementary Table 1. Quality assessment of studies | | | |
| --- | --- | --- | --- |
| Quality Assessment of Cross-section Studies | | | |
| Study, year | score | Study | score |
| Zakopoulos1999 | 6 | Grandi,A.M2001 | 6 |
| Nakashima2004 | 7 | Kuwajima,I1993 | 6 |
| Manios,E2016 | 8 | Yildirim,N2002 | 7 |
| Kamel,N2006 | 7 | Pose-Reino,A1996 | 6 |
| Muldoon2000 | 7 | Torrisi,G1999 | 6 |
| Shih-Hsien2013 | 6 | Ermis,N2016 | 6 |
| Androulakis,E2016 | 7 | Sega,R2001 | 7 |
| Vyssoulis2010 | 7 | Soma1996 | 6 |
| Longo,D 2006 | 7 | Sang-Hyun2009 | 7 |
| Andrikou,I2011 | 7 | Mustafa2013 | 7 |
| Neil,J.Wimmer2007 | 6 | Verdecchia1993 | 6 |
| Quality Assessment of Cohort Studies | | | |
| Study | Selection | Comparability | Exposure |
| Polonia2005 | **★★★★** | ★ | ★★★ |
| Hoshide2002 | **★★★★** | ★★ | **★★★** |
| R. H. Fagard2000 | **★★★** | ★ | **★★** |
